# Supplementary material for: Decomposing cortical activity through neuronal tracing connectome-eigenmodes in marmosets
Source: Nat Commun. 2024 Mar 13;15:2289. doi: 10.1038/s41467-024-46651-8 (PMC10937940; doi:10.1038/s41467-024-46651-8)
Supplement: Supplementary file 1 — Supplementary Information [file 41467_2024_46651_MOESM1_ESM.pdf]

Supplementary information for

Decomposing Cortical Activity through Neuronal Tracing Connectome-eigenmodes in Marmosets

Jie Xia[1,2], Cirong Liu[3], Jiao Li[1,2], Yao Meng[1,2], Siqi Yang[4], Huafu Chen[1,2]✉, Wei Liao[1,2]✉

[1] The Clinical Hospital of Chengdu Brain Science Institute, School of Life Science and Technology, University of Electronic Science and Technology of China, Chengdu 611731, P.R. China.
[2] MOE Key Lab for Neuroinformation, High-Field Magnetic Resonance Brain Imaging Key Laboratory of Sichuan Province, University of Electronic Science and Technology of China, Chengdu 611731, P.R. China.
[3] Institute of Neuroscience, CAS Key Laboratory of Primate Neurobiology, Center for Excellence in Brain Science and Intelligence Technology, Chinese Academy of Sciences, Shanghai 200031, P.R. China.
[4] School of Cybersecurity, Chengdu University of Information Technology, Chengdu 610225, P.R. China.

✉ Corresponding authors:
Huafu Chen (chenhf@uestc.edu.cn), and Wei Liao (weiliao.wl@gmail.com).
The Clinical Hospital of Chengdu Brain Science Institute, MOE Key Laboratory for Neuroinformation, University of Electronic Science and Technology of China, Chengdu 611731, P.R. China. Fax: +86-28-61831273. Tel: +86-28-61831273.

Table of contents

Supplementary Methods 3

S1 MRI data in marmosets 3

S1.1 Data acquisition 3

S1.2 Data preprocessing 3

S2 Graph signal processing on the marmoset data 4

S2.1 Normalized directed graph Laplacian 4

S2.2 Generation of graph surrogate signals 6

S3 Connectivity embedding 7

S4 Spatial correction for brain map similarity 7

Supplementary Results 8

S5 Verify the ergodicity of the transition probability matrix 8

S6 Sensitivity analyses 9

S7 Localized eigenmodes can reconstruct activity patterns in marmosets 10

Supplementary Figures 11

Supplementary Tables 26

Supplementary References 30

Supplementary Methods

S1 MRI data in marmosets

S1.1 Data acquisition

Seven marmosets (all male; 3–9 years) from the National Institutes of Health (NIH) cohort were scanned using a 7T/30 cm horizontal MRI[1,2]. Blood oxygen level-dependent functional MRI (BOLD-fMRI) data were collected in ParaVision 6.0.1 software using a 2D gradient echo planar imaging (EPI) sequence (TR = 2000 ms, TE = 22.2 ms, flip angle = 70.4°, FOV = 28 × 36 mm^2, matrix size = 56 × 72, 38 axial slices, resolution = 0.5 mm isotropic, 512 time points, each run is 17 min long). Two sets of spin-echo EPI with opposite phase-encoding directions (LR and RL) were also collected for EPI-distortion correction (TR = 3000 ms, TE = 36 ms, flip angle = 90°, FOV = 28 × 36 mm°, matrix size = 56 × 72, 38 axial slices, slice thickness = 0.5 mm, 8 volumes for each set). For each session, a T2-weighted structural image was scanned for spatial registration (TR = 6000 ms, TE = 9 ms, flip angle = 90°, FOV = 28 × 36 mm^2, matrix size = 112 × 144, 38 axis slices, slice thickness=0.5 mm). Furthermore, multishell diffusion-weighted imaging (DWI) was collected using a 2D spin-echo EPI sequence as follows: TR = 5100 ms, TE = 38 ms, the number of segments = 88, FOV = 36 × 28 mm^2, matrix size = 72 × 56, slice thickness = 0.5 mm, a total of 400 DWI images for two-phase encodings (blip-up and blip-down) and each has 3 b values (8 *b*= 0, 64 *b* = 2400, and 128 *b*= 4800), and the scanning duration was ~34 min.

S1.2 Data preprocessing

All fMRI data were preprocessed using AFNI (v18.0.11, https://afni.nimh.nih.gov/)[3],

FSL (v5.1, https://fsl.fmrib.ox.ac.uk/fsl/fslwiki/)[4], ANTs (v2.1, https://picsl.upenn.edu/software/ants/)[5], and Connectome Workbench (v1.5.0, https://www.humanconnectome.org/software/connectome-workbench)[6]. In brief, BOLD-fMRI data underwent minimal preprocessing procedures, which involved slice-timing correction (AFNI's *3dTshift*), motion correction (AFNI's *3dvolreg*), EPI distortion correction (FSL's *topup*). Then, several regression covariates were considered to remove potential noise and motion effects (AFNI's *3dDeconvolve*), including demeaned and derivatives of motion parameters, motion-censor regressors, white matter, and cerebrospinal fluid signal. Next, the BOLD-fMRI data was band-pass filtered, corresponding to the frequency range 0.01 to 0.1 Hz (AFNI's *3dTproject*). Furthermore, the preprocessed fMRI data was registered to its T2-weighted image using rigid-body transformation. The T2-weighted images were then nonlinearly transformed to the template space of Marmoset Brain Atlas Version-3 (MBMv3, https://marmosetbrainmapping.org/v3.html)[7]. All preprocessed fMRI data were mapped to 3D brain surfaces of the MBMv3 using the Connectome Workbench. Finally, fMRI data was smoothed using a 1 mm full-width at half-maximum (FWHM) Gaussian kernel to reduce noise.

S2 Graph signal processing on the marmoset data

S2.1 Normalized directed graph Laplacian

A random walk defined on a strongly connected directed weighted graph $\mathcal{G} = (V, E, A)$ is a homogeneous Markov chain $\chi = (X_n)_{n\geq 0}$ with a finite state space. Its transition probability is proportional to the weight of the edge[8]. The random walk is defined by a transition probability matrix P, P is defined as, $P(i,j) = \frac{A_{ij}}{\sum_j A_{ij}}$, where $P(i,j)$ denotes the probability of walking from node i to node j. Since the entries of

$P(i,j)$ are probabilities, it follows that $P(i,j) \geq 0$, and $\sum_{j \in V} P(i,j) = 1, \forall\ i,j \in V$. From the point of view of graph theory, the transition matrix $P \in R^{N \times N}$[9,10] is equal to

$$P = D^{-1}A, \tag{1}$$

where $D = D_{ii} = \sum_{j=1}^{N} A_{ij}$, denotes the diagonal matrix of the out-degrees of the adjacency matrix A.

For a strongly connected directed graph $\mathcal{G}$, the Markov chain χ with transition matrix P is ergodic (irreducible and aperiodic), then it has a unique stationary distribution π (Perron vector), i.e. $\pi P = \pi$, with $\sum_{i=1}^{N} \pi(v_i) = 1, \pi(v_i) \geq 0$[11,12]. The normalized Laplacian of a directed graph $\mathcal{G}$[8] is defined by,

$$\mathcal{L} = I - \frac{\Pi^{1/2} P \Pi^{-1/2} + \Pi^{-1/2} P^T \Pi^{1/2}}{2}, \tag{2}$$

where $\Pi = diag\{\pi(v_1), \cdots, \pi(v_N)\}$ is the diagonal matrix of the stationary distribution π, I is the identity matrix, and P^T means for the transpose of P. The Laplacian satisfies $\mathcal{L}^T = \mathcal{L}$. See Supplementary Algorithm 1 for pseudocode. An example of computing normalized digraph Laplacian $\mathcal{L}$ is shown in Supplementary Fig. 3.

Supplementary Algorithm 1: Normalized directed graph Laplacian

Input: $A \in R_{+}^{N \times N}$: directed weighted adjacency matrix

Output: L: normalized directed graph Laplacian

1: Compute the diagonal matrix D of the out-degrees of A.

2: Compute the transition matrix P of the random walk, see Eq.(1).

3: Compute the stationary distribution π, i.e., $\pi P = \pi$, with $\sum_{i=1}^{N} \pi(v_i) = 1$, $\pi(v_i) \geq 0$.

π can be obtained by calculating the Perron vector of P, which can be calculated by using the Matlab function "*eigs*".

4: Calculate the diagonal matrix $\Pi = diag\{\pi(v_1), \cdots, \pi(v_N)\}$ of the stationary distribution π,

5: Calculate the normalized directed graph Laplacian L, see Eq. (2),

6: Return a symmetric matrix L.

S2.2 Generation of graph surrogate signals

We generated three types of graph surrogate signals.

Rewired connectome. The original connectome was randomized into a rewired network using the Brain Connectivity Toolbox (https://sites.google.com/site/bctnet/)[13]. The procedure destroyed the topological organization of the original connectome while preserving its degree, weight, and strength distributions[14]. Then, cortical activity was projected onto eigenmodes of these rewired networks to reconstruct the surrogate activity patterns.

Moran spectral randomization. The spatially constrained surrogate cortical activity was generated by the Moran spectral randomization (MSR) approach[15]. This method was initialized with eigenvectors of inverse distance matrix between brain regions. The surrogate cortical activity was performed using the singleton procedure implemented in BrainSpace (http://github.com/MICA-MNI/BrainSpace)[16]. Then, the surrogate cortical activity was projected onto eigenmodes of the original connectome to reconstruct the activity patterns.

Graph spectral randomization. The graph surrogate signals were generated using the graph spectral randomization procedure[17-19]. The approach used sign-randomization to generate the graph spectral coefficients while preserving the original connectome architecture. Assuming that the random sign flips were stored on the diagonal of Φ_{graph}. The eigenmodes of empirical connectome were replaced by spectral randomization $\Psi\Phi_{graph}$ to generate the surrogate signals,

$$f_{v_i}^{(null)}(t) = \Psi\Phi_{graph}\Psi^T f_{v_i}(t) \tag{3}$$

Graph spectral randomization surrogate signals preserved the empirical energy spectral density but destroyed specific interactions among eigenmodes as represented

by empirical signals[19].

S3 Connectivity embedding

We used diffusion embedding[20] to project a set of embeddings into a lower-dimensional space. Briefly, we used the top 10% connections of each row of the FC matrix to compute non-negative symmetric affinity matrices that capture the similarity between features in cortical areas. Diffusion map embedding was used to estimate low-dimensional representations of affinity matrices. This algorithm was controlled by two parameters α and t. We followed the previous recommendation[20-22] and set α = 0.5 and t = 0, which preserved the global relationship between data points in the embedding space.

S4 Spatial correction for brain map similarity

The intrinsic spatial smoothing of two given brain maps may exaggerate the significance of their spatial correlation. To this end, we performed a permutation test to generate spatial autocorrelation (SA)-preserving surrogate brain maps using variogram matching[23,24]. The parametric method can be subdivided into two main steps. First, the empirical brain map is randomly permuted. Then, the permuted maps are smoothed and rescaled to reintroduce the SA characteristic of the empirical brain map. This approach can be implemented through the brainSMASH software (https://github.com/murraylab/brainsmash)[23]. Specifically, we used BrainSMASH to generate 1000 surrogate maps of the empirical map. The p_{SMASH} value was calculated as the percentile rank of the surrogate correlations that are more extreme than the observed correlation (<5th or >95th percentile).

Supplementary Results

S5 Verify the ergodicity of the transition probability matrix

We defined a Markov chain $\chi = (X_n)_{n\geq 0}$ with transition matrix P, $P = D^{-1}A$, where $D = \sum_{j=1}^{N} A_{ij}$ denotes the out-degrees matrix of the adjacency matrix A, $A = \{A_{ij}\}_{1\leq i,j\leq N} \in R_{+}^{N\times N}$ is defined as the FLNe value of cellular connectome (CC) in marmosets.

A Markov chain χ is ergodic if it is both irreducibile and aperiodic[25]. This condition is equivalent to the transition matrix being a primitive non-negative matrix. By Wielandt's theorem[26], a Markov chain χ is ergodic if and only if all elements of P^s are positive for $s = (N-1)^2 + 1$, where N is the number of nodes of a directed graph. We used the Matlab function "*isergodic*" to determine the ergodicity of the Markov chain χ (https://ww2.mathworks.cn/help/econ/dtmc.isergodic.html). Visually confirm that the Markov chain χ is ergodic by plotting its eigenvalues on the complex plane (Supplementary Fig. 14).

By the Perron-Frobenius Theorem[27], if the Markov chain χ is ergodic with a diagonalizable transition matrix P, the diagonalization of P admits a simple dominant eigenvalue $\lambda_{max} = 1$, and an accompanying non-negative left eigenvector that normalizes to a unique stationary distribution. All other eigenvalues have a modulus less than 1, which means that all eigenvalues different from λ_{max} lie within the unit circle[11]. Therefore, we verified that the Markov chain χ with transition matrix P defined on a directed graph $\mathcal{G} = (V, E, A)$ is ergodic (irreducible and aperiodic).

Furthermore, we used the Matlab function "*eigs*" to calculate the stationary distribution (Perron vector) π of the transition matrix P of Markov chain χ (Supplementary Fig.15). The stationary distribution reflects that the distribution of

states converges to a stable distribution as the Markov chain progresses, which provides insights into the system's long-term behavior.

S6 Sensitivity analyses

We reported results representing the low- and high-frequency components using median-split frequency $C(C = 12)$ based on the graph spectrum dichotomy approach[19]. To verify the robustness of the filter cut-off setting, we decomposed the observed BOLD-fMRI signals into low- and high-frequency components using $K_L = 7$ to $K_L = 11$, and $K_L = 13$ to $K_L = 17$ (K_L from five below to five above the cut-off frequency C in the main text), and K_L ranging from $K_H = 31$ to $K_H = 40$. We then computed the spatial correlations of low- and high-frequency components, as well as the CFD patterns between the original and robustness analyses.

We found that the observed signal decomposition was robust to the choice of filter cut-off parameter: for low-frequency components (Supplementary Fig. 8a), the spatial correlation values ranged from 0.785 to 0.942, all p_{SMASH} < 0.0001, and the mean spatial correlation was 0.868 (S.D. = 0.066); for high-frequency components (Supplementary Fig. 8b), the spatial correlation values ranged from 0.647 to 0.852, all P_{SMASH} < 0.0001, and the mean spatial correlation was 0.747 (S.D.= 0.068).

Furthermore, we found that the CFD patterns were consistent across the filter cut-off setting (Supplementary Fig. 9): the spatial correlation coefficients ranged from 0.779 to 0.920, all p_{SMASH} < 0.0001, and the mean spatial correlation was 0.872 (S.D.= 0.051).

In summary, low- and high-frequency components, as well as the CFD patterns are highly stable regardless of over the filter cut-off setting.

S7 Localized eigenmodes can reconstruct activity patterns in marmosets

To examine whether localized cellular connectome (CC) eigenmodes can reconstruct activity patterns in marmosets, we used the same reconstruction framework to reconstruct cortical activity by a basis set derived from the CC matrix of homologous areas (Supplementary Fig. 12a). As the eigenvalue increased, the spatial distribution of homologous CC eigenmodes became more intricate (Supplementary Fig. 12b). We found that increasing homologous CC eigenmodes enhanced the reconstruction accuracy of marmoset cortical activity concentration (Supplementary Fig. 12c) and FC (Supplementary Fig. 12d). The first five CC eigenmodes reached 86% and 83% reconstruction accuracy of cortical activity concentration and FC in marmosets, respectively. Reconstructing marmoset cortical activity concentration and FC using homologous CC eigenmodes showed better performance than rewired CC connectomes ($p_{\text{rewired}} < 0.001$, FDR-corrected) and randomly chosen non-homologous area' cortical activity ($p_{\text{perm}} < 0.001$, FDR-corrected). These results suggest that eigenmodes anatomically localized can reconstruct brain activity patterns[28], which is consistent with the classic neuroscientific paradigm in which cortical activity should be described as "discrete, isolated, and anatomically localized" clusters[29].

Supplementary Figures

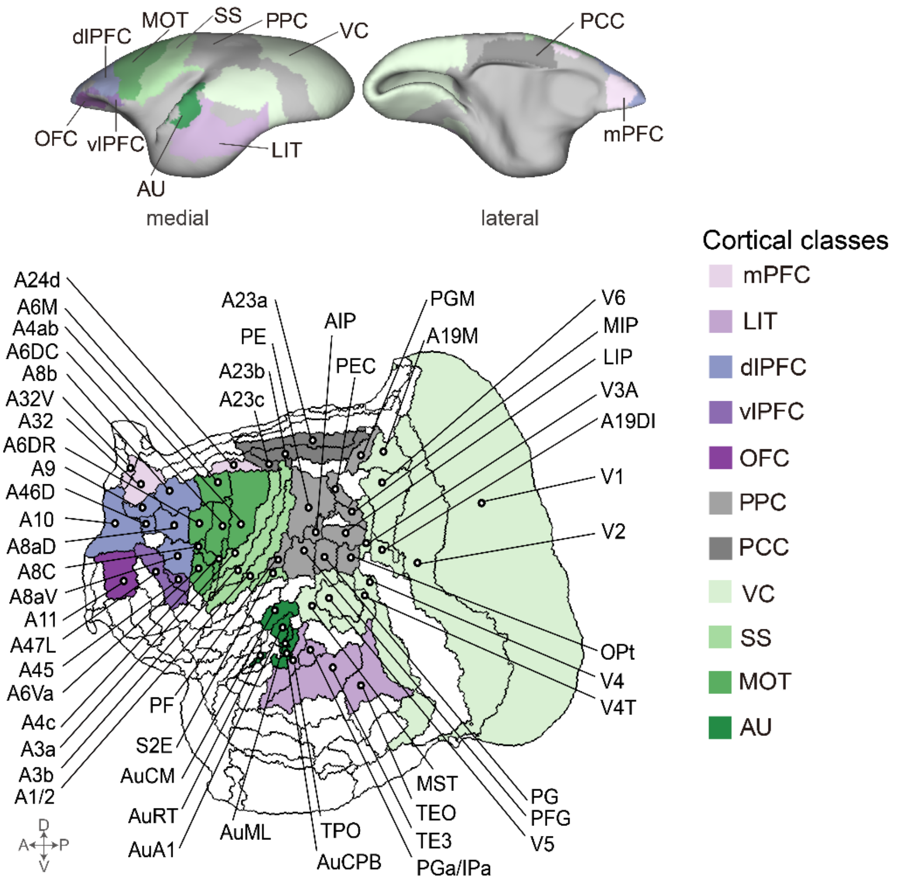

Fig. S1. The flat map of marmosets. The analysis focused on 55 cortical areas marked with different colors on a two-dimensional flat map of the marmoset cortex according to the different cortical classes[7,30]. The white-shaded areas were those for which no tracer was injected. For abbreviations of the brain areas, see Supplementary Table 1. AU, auditory cortex; dlPFC, dorsolateral prefrontal cortex; LIT, lateral and inferior temporal cortex; mPFC, medial prefrontal cortex; MOT, motor and premotor cortex; OFC, orbitofrontal cortex; PCC, posterior cingulate, medial and retrosplenial cortex; PPC, posterior parietal cortex; SS, somatosensory cortex; vlPFC, ventrolateral prefrontal cortex; VC, visual cortex.

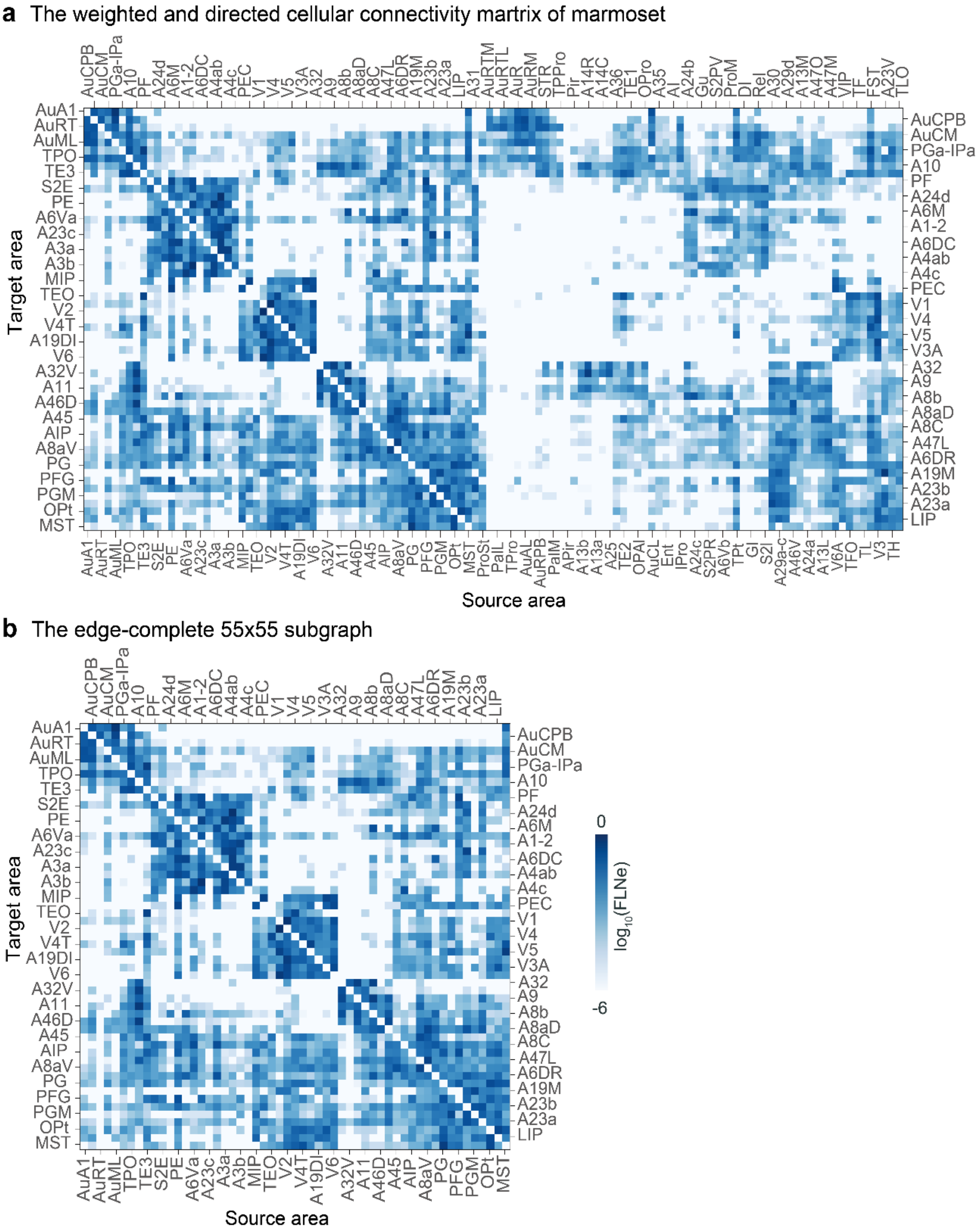

Fig. S2. Neuronal tracing cellular connectivity (CC) matrix of marmosets[31]. a A weighted and directed CC matrix of marmosets. The rows were the 55 target areas, and the columns were the 116 source areas that provided inputs to the target areas. Each entry in the matrix was the base 10 logarithm of the arithmetic mean of the FLN (log_{10}(FLNe)) across injections within the same target area. Darker shades of color represented stronger connection weights, and white represented no connection. **b** The edge-complete subgraph, in which all inputs and outputs were known, showed a dense matrix of topological connections.

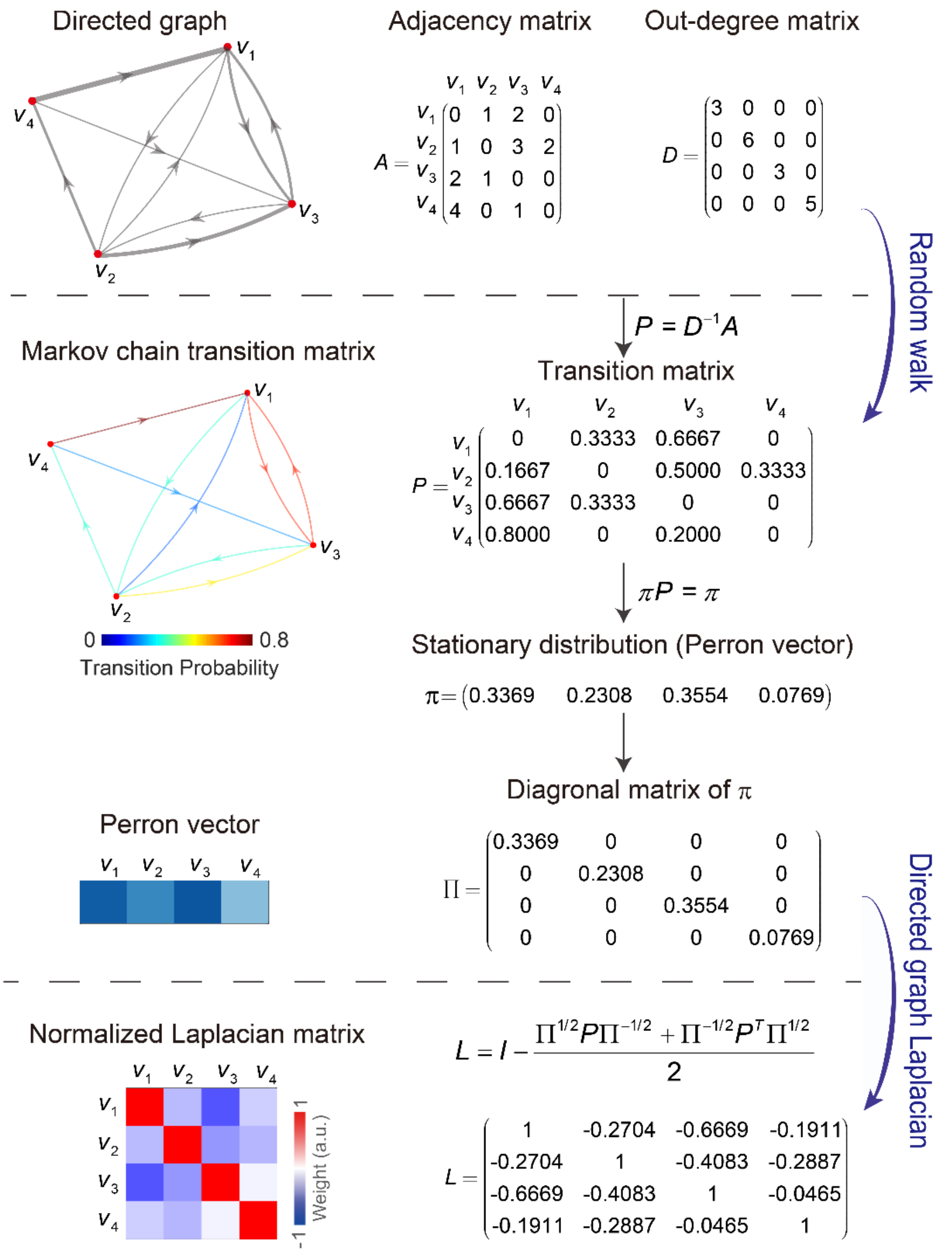

Fig. S3. An example of computing normalized digraph Laplacian. First, a directed graph is represented as an asymmetric weighted adjacency matrix. Second, compute the diagonal matrix D of the out-degrees of A. Third, compute the transition matrix P of the random walk $\chi = (X_n)_{n \geq 0}$. Fourth, compute the stationary distribution π, which can be obtained by calculating the Perron vector of P. Finally, calculate the normalized directed graph Laplacian L.

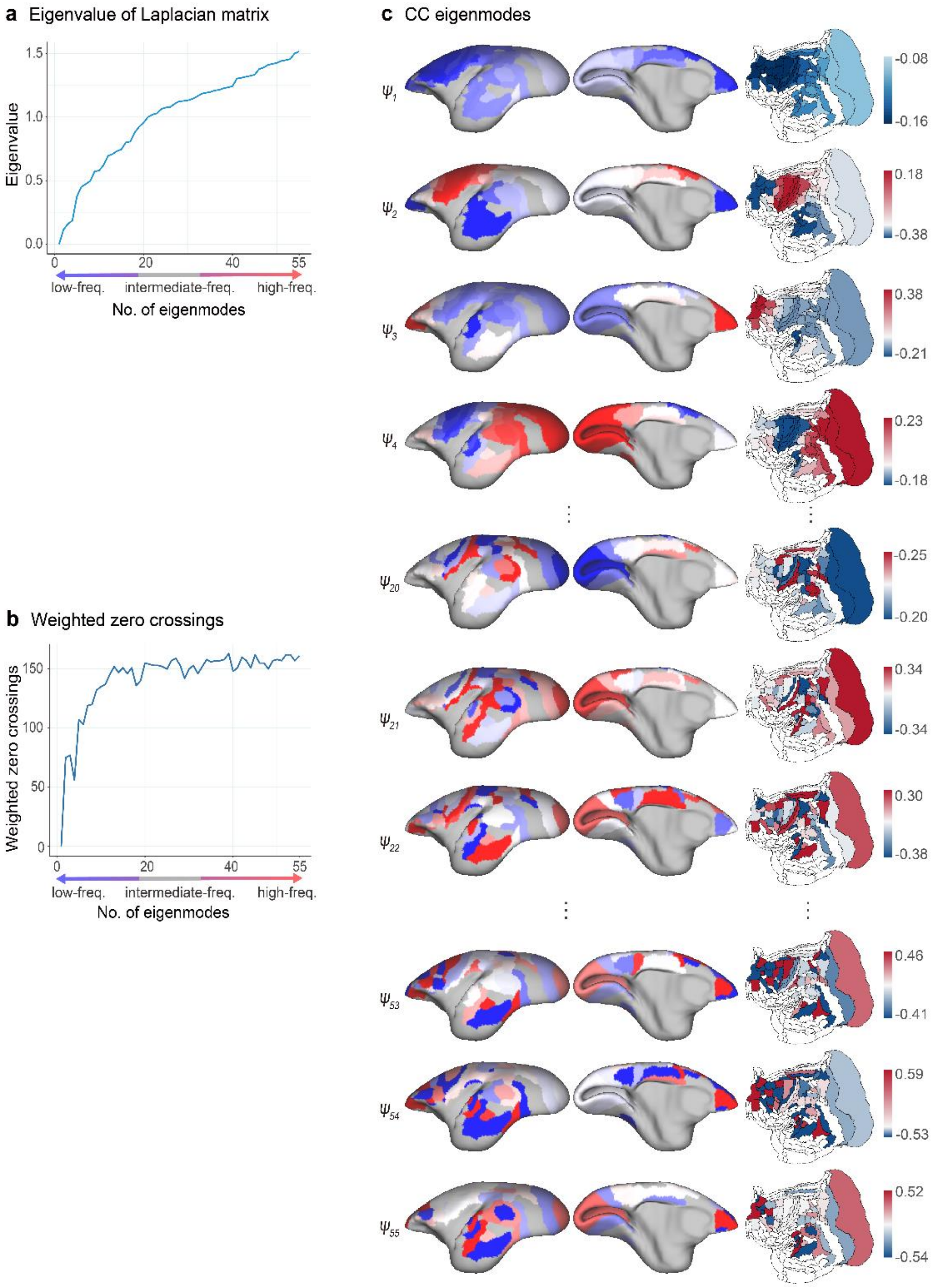

Fig. S4. Cellular connectome (CC) eigenmodes. a The eigenvalues of the Laplace matrix. **b** The number of weighted zero crossings along the graph structure for each CC eigenmode. The Laplacian eigenvectors associated with larger eigenvalues cross zero more often, suggesting the increasing complexity of eigenmodes with higher spatial frequency. **c** Several CC eigenmodes were projected onto the marmoset brain surface or flat map. Blue–white–red colors represented negative–zero–positive values. We showed the four eigenmodes corresponding to the four smallest graph Laplacian eigenvalues (low-frequency eigenmodes), the three eigenmodes corresponding to the middle of eigenvalues (intermediate-frequency eigenmodes), and the three eigenmodes corresponding to the three largest eigenvalues (high-frequency eigenmodes).

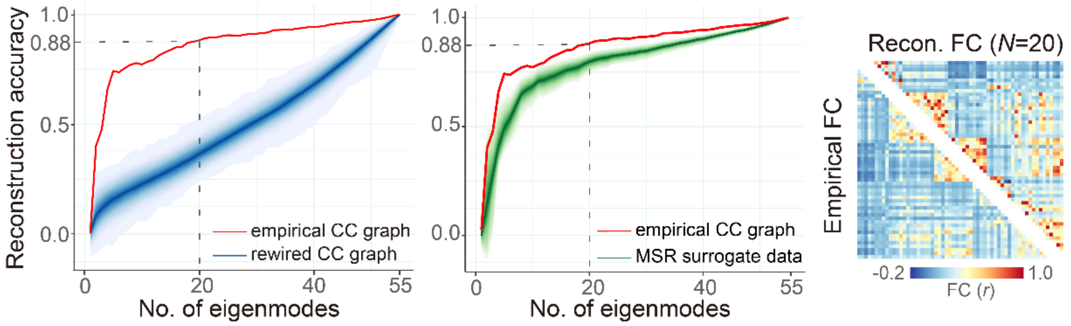

Fig. S5. Reconstruction accuracy of marmoset functional connectivity (FC) achieved by cellular connectome (CC) eigenmodes. Reconstruction accuracy was quantified as the Pearson correlation between the empirical and reconstructed FC matrices across edges. The solid line indicates the reconstruction accuracy of the empirical CC graph. The shading lines (left) indicate the reconstruction accuracy using eigenmodes derived from rewired CC graphs (1000 repetitions) to reconstruct unperturbed activity. The shading lines (middle) indicate the reconstruction accuracy using empirical CC eigenmodes to reconstruct the Moran spectral randomization (MSR) surrogate cortical activity (1000 repetitions). The shading indicates the 95th percentile interval of the null distributions.

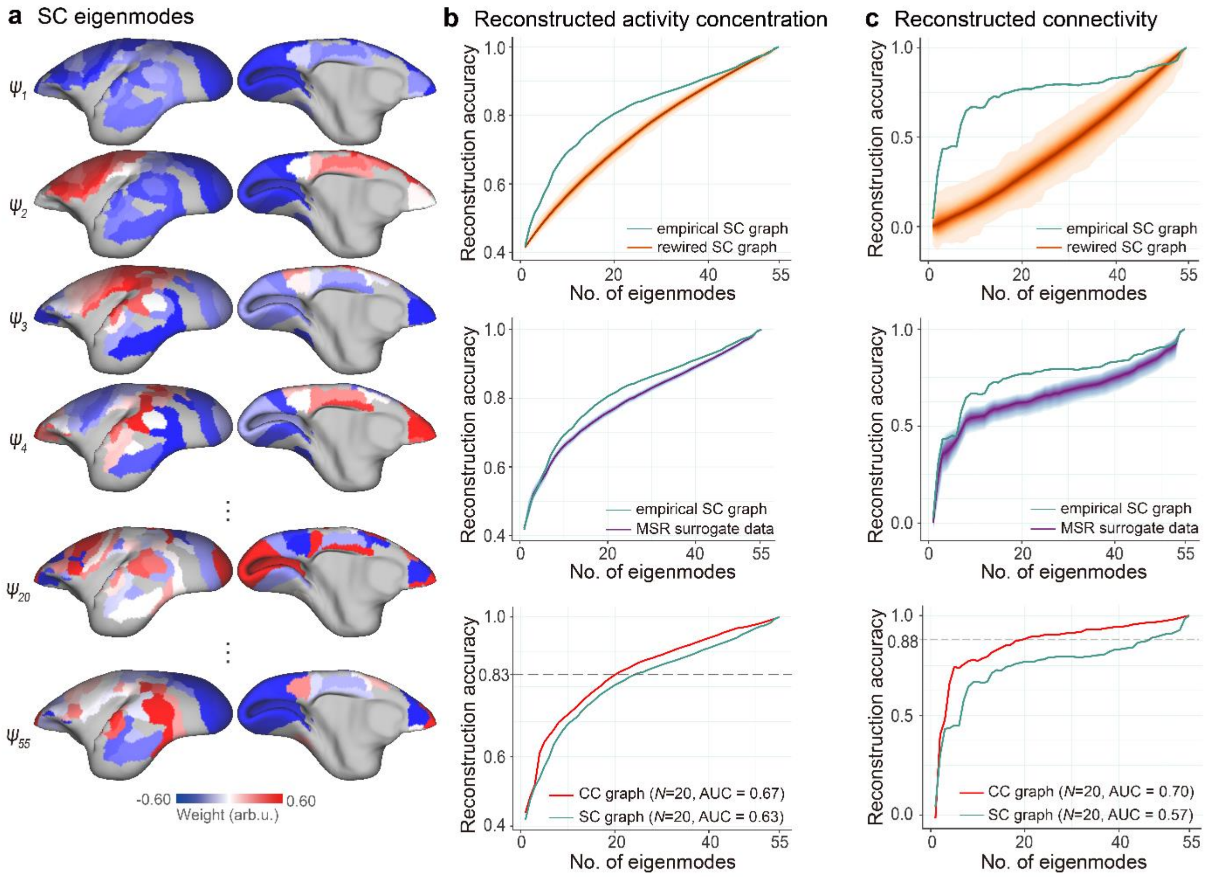

Fig. S6. Cellular connectome (CC) eigenmodes were compared to structural connectome (SC) eigenmodes. a The parts of SC eigenmodes were projected onto the marmoset brain surface. Blue–white–red colors represented negative–zero–positive values. **b** Reconstruction accuracy of marmoset cortical activity concentration (L_2-norm across time points) achieved by SC and CC eigenmodes. Reconstruction accuracy was quantified as the ratio between empirical and reconstructed cortical activity concentration. **c** Reconstruction accuracy (i.e., Pearson correlation coefficients between empirical and reconstructed FC matrices across edges) of marmoset FC achieved by SC and CC eigenmodes. The solid line indicates the reconstruction accuracy of the empirical SC graph. The shading lines (upper) indicate the reconstruction accuracy using eigenmodes derived from rewired SC graphs (1000 repetitions) to reconstruct unperturbed activity. The shading lines (middle) indicate the reconstruction accuracy using empirical SC eigenmodes to reconstruct the Moran spectral randomization (MSR) surrogate cortical activity (1000 repetitions). The shading indicates the 95th percentile interval of the null distributions. The solid line (bottom) indicates the reconstruction accuracy of the empirical CC graph.

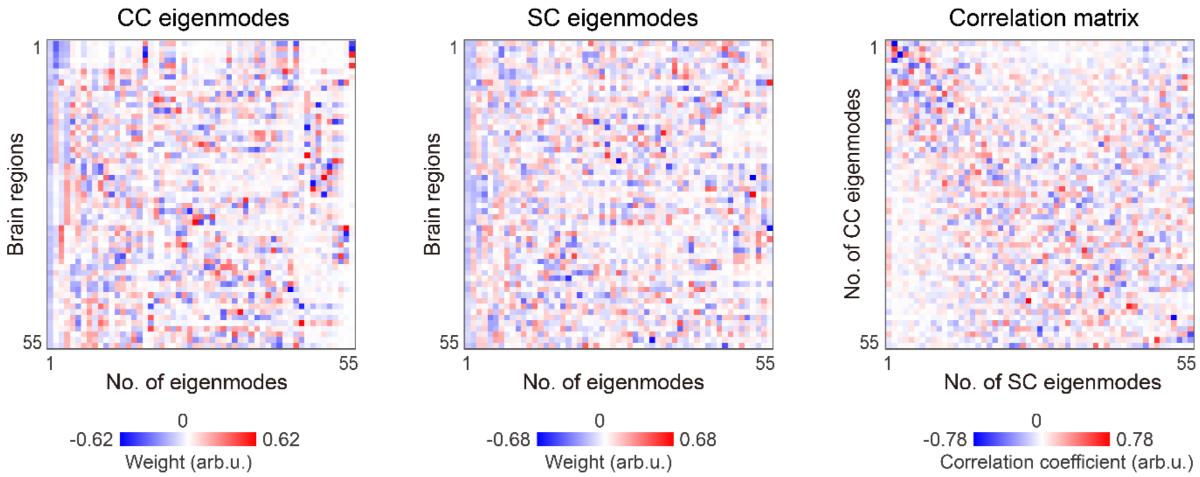

Fig. S7. The correlation matrix between cellular connectome (CC) eigenmodes and structural connectome (SC) eigenmodes. We calculated the Pearson correlation to quantify the spatial pattern difference between CC and SC eigenmodes. Bluer and redder colors represent CC and SC eigenmodes with similar spatial distribution.

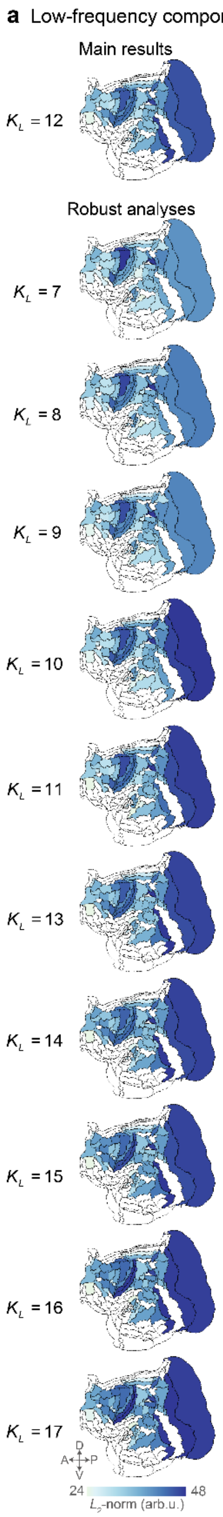

K_L	ρ	p_{SMASH}
7	0.812	<0.0001
8	0.888	<0.0001
9	0.884	<0.0001
10	0.915	<0.0001
11	0.980	<0.0001
13	0.942	<0.0001
14	0.863	<0.0001
15	0.806	<0.0001
16	0.800	<0.0001
17	0.785	<0.0001

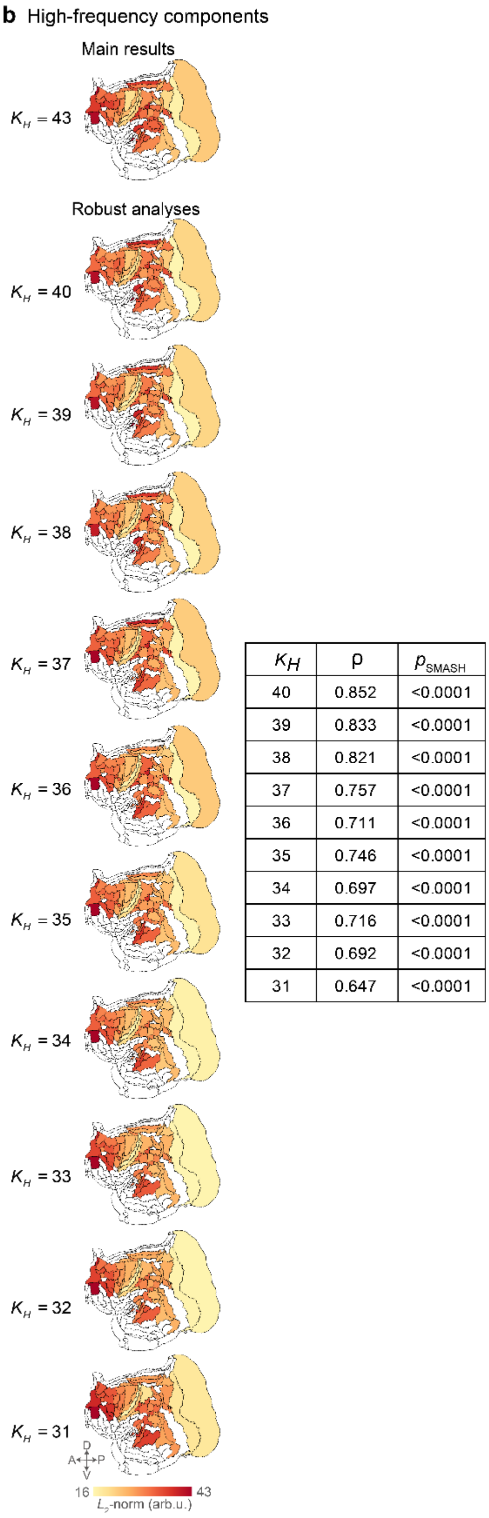

K_H	ρ	p_{SMASH}
40	0.852	<0.0001
39	0.833	<0.0001
38	0.821	<0.0001
37	0.757	<0.0001
36	0.711	<0.0001
35	0.746	<0.0001
34	0.697	<0.0001
33	0.716	<0.0001
32	0.692	<0.0001
31	0.647	<0.0001

Fig. S8. Robustness of low- (a) and high-frequency (b) components to filter cut-off setting. The observed signal decomposition was robust under different low- and high-frequency eigenmodes. The significance (p_{SMASH}) of Spearman's correlation coefficients (ρ) is evaluated using spatial autocorrelation-preserving surrogate brain maps (1000 repetitions, two-sided).

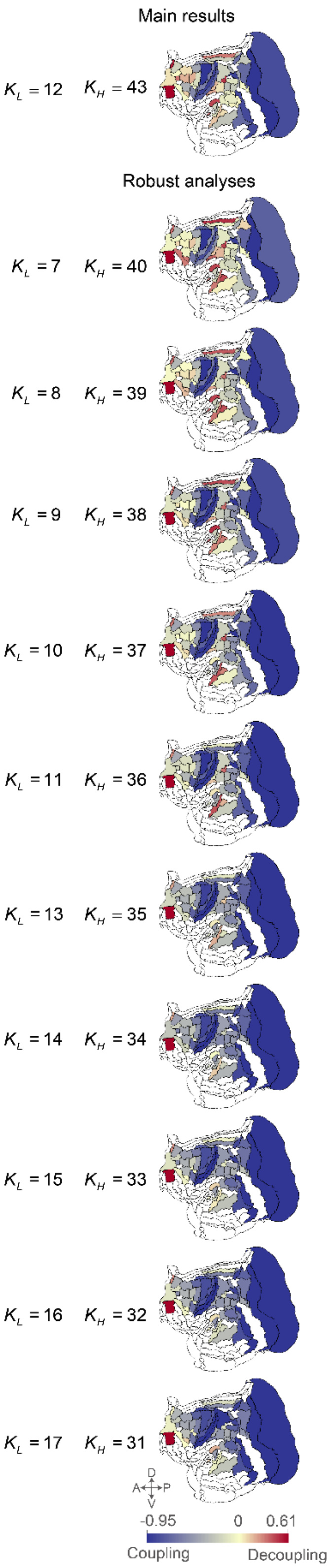

K_L	K_H	ρ	p_{SMASH}
7	40	0.912	<0.0001
8	39	0.920	<0.0001
9	38	0.907	<0.0001
10	37	0.901	<0.0001
11	36	0.918	<0.0001
13	35	0.893	<0.0001
14	34	0.845	<0.0001
15	33	0.827	<0.0001
16	32	0.815	<0.0001
17	31	0.779	<0.0001

Fig. S9. Robustness of regional cellular-functional decoupling (CFD) pattern to filter cut-off setting. The patterns of CFD were stable under different low- and high-frequency eigenmodes. The significance (p_{SMASH}) of Spearman's correlation coefficients (ρ) is evaluated using spatial autocorrelation-preserving surrogate brain maps (1000 repetitions, two-sided).

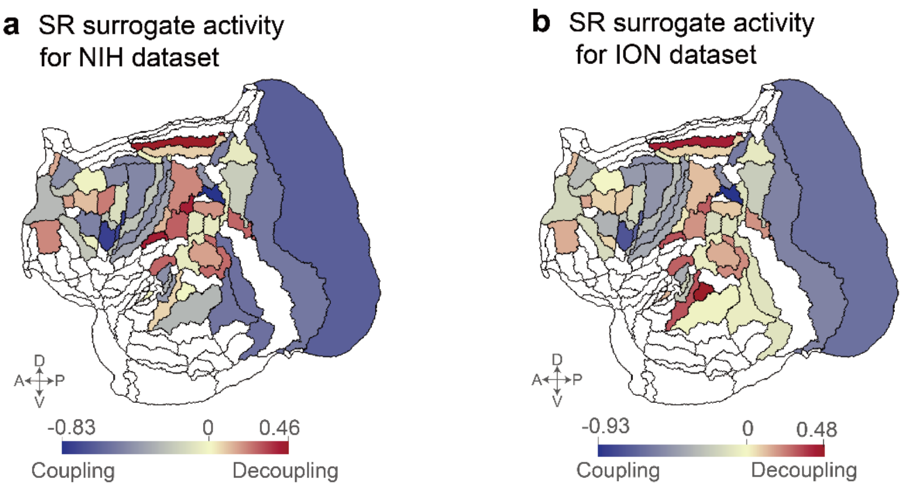

Fig. S10. The cellular-function decoupling (CFD) patterns for the National Institutes of Health (NIH) dataset (a) and the Institute of Neuroscience (ION) dataset (b) of graph spectral randomization (SR) surrogate activity. The surrogate brain activity time courses were built as a linear combination of neuronal tracing connectome eigenmodes with randomized coefficient signs. The surrogate brain activity generated a null distribution of the CFD index, thus allowing the detection of significantly coupled or decoupled areas in empirical brain activity.

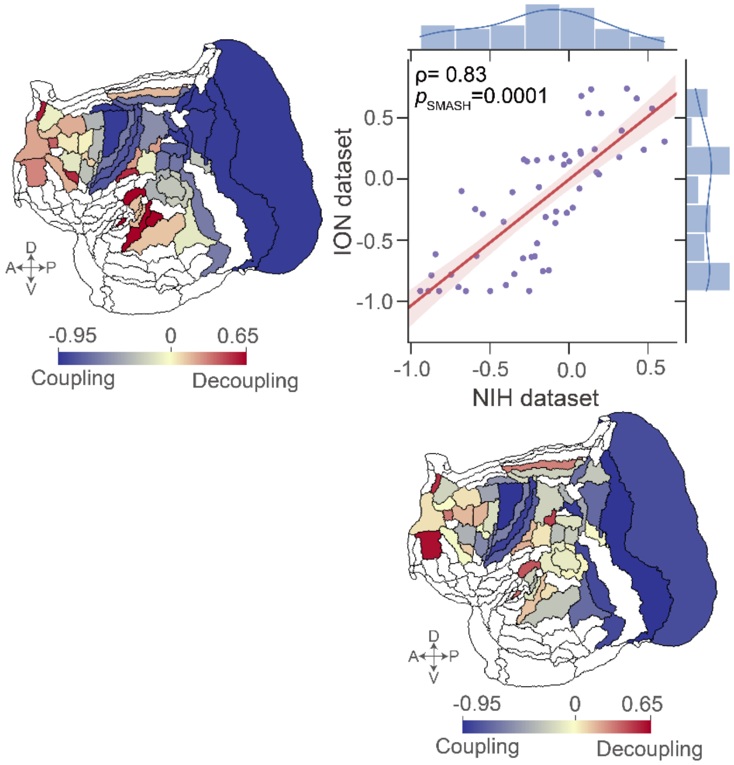

Fig. S11. Repeatability analysis of regional cellular-functional decoupling (CFD) pattern in two independent datasets. The spatial correlation of the CFD pattern (n = 55 brain areas) between the National Institutes of Health (NIH) sites dataset and the Institute of Neuroscience (ION) sites dataset showed excellent reproducibility. The regression lines are shown for both relationships. Shaded bands represent the 95% confidence intervals; and histograms correspond to each variable. The significance (p_{SMASH}) of Spearman's correlation coefficients (ρ) is evaluated using spatial autocorrelation-preserving surrogate brain maps (1000 repetitions, two-sided).

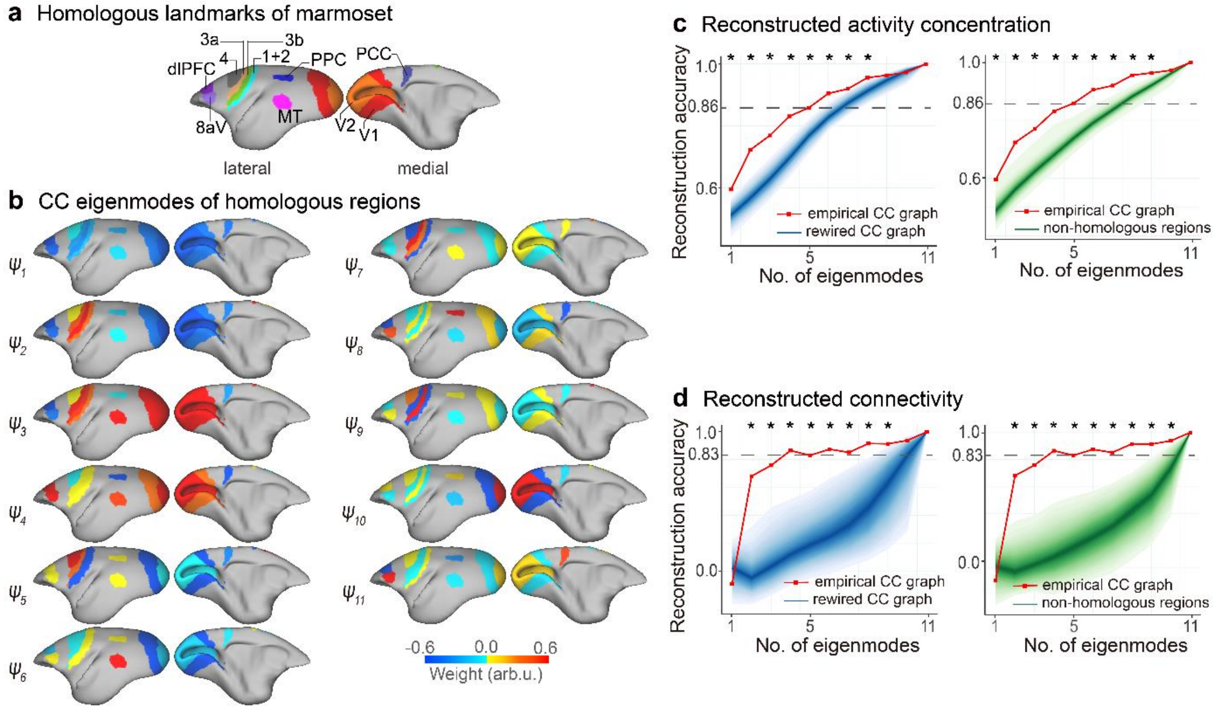

Fig. S12. Reconstruction accuracy of marmoset cortical activity concentration and functional connectivity (FC) achieved by homologous cellular connectome (CC) eigenmodes. a Homologous landmarks (*n* = 11 brain areas) in marmosets. **b** The CC eigenmodes of homologous regions. **c** Reconstruction accuracy of marmoset cortical activity concentration (L_2-norm across time points) across 11 common homologous areas. **d** Reconstruction accuracy of marmoset FC. The solid line indicates the reconstruction accuracy of the empirical CC graph of homologous regions. The shading lines (left) indicate the reconstruction accuracy using eigenmodes derived from rewired CC graphs (1000 repetitions) to reconstruct unperturbed activity. The shading lines (right) indicate the reconstruction accuracy using homologous CC eigenmodes to reconstruct randomly selected non-homologous cortical activity (1000 repetitions). The observed reconstruction accuracy (solid lines) was compared to the accuracy obtained from rewired CC connectomes (shading lines) and randomly selected non-homologous areas' cortical activity (shading lines). The shading indicates the 95th percentile interval of the null distributions. Asterisks denote a statistically significance level at $p < 0.05$ (one-sided, FDR-corrected).

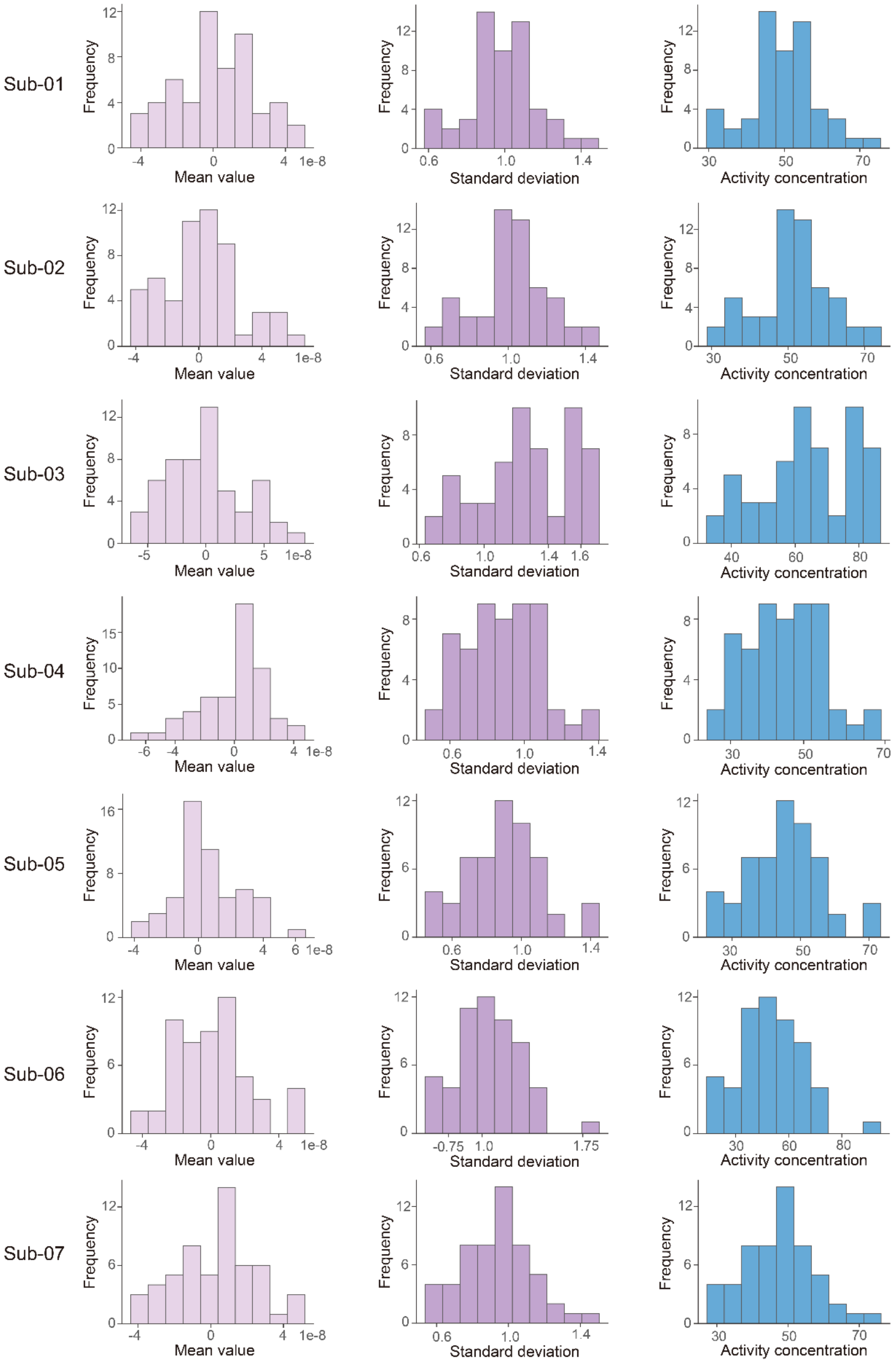

Fig. S13. The mean, standard deviation, and activity concentration distribution of regional BOLD-fMRI signals. Regional activity concentration was defined as the L_2-norm of BOLD-fMRI signals across all time points.

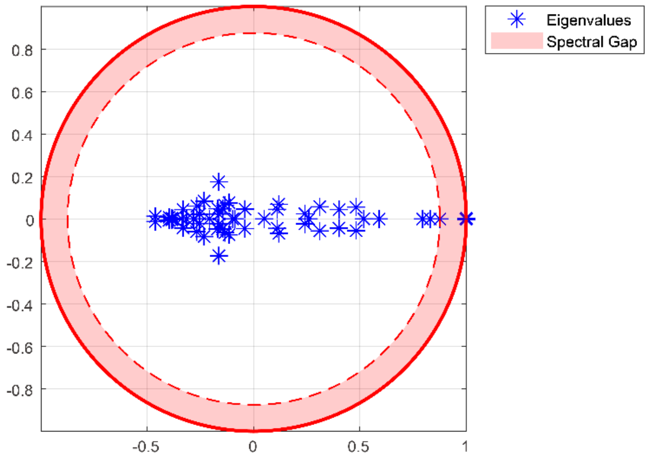

Fig. S14. The eigenvalues of the transition matrix of Markov chain χ. The Matlab function "*eigplot*" plots the eigenvalues of the transition matrix P. The plot highlights the unit circle, the Perron-Frobenius eigenvalue at (1,0), the second largest eigenvalue magnitude circle, and the spectral gap between the two circles.

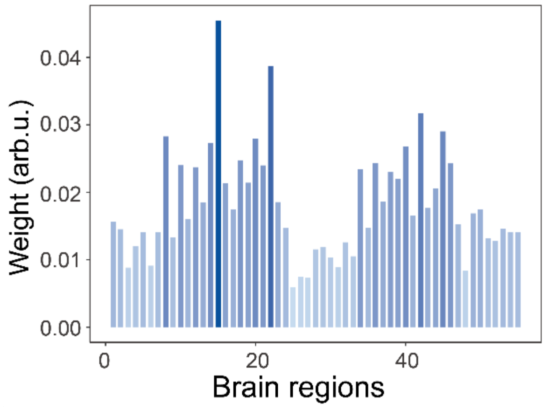

Fig. S15. Visualization stationary distribution (Perron vector) π of the transition matrix P. The Perron vector π of P can be calculated using the Matlab function "*eigs*".

Supplementary Tables

Table S1. Abbreviations of the names of cortical areas grouped in the major cortical subdivisions[7,30].

Abbreviation	Full name
**Dorsolateral prefrontal cortex (dlPFC)**	
A10	Area 10
A9	Area 9
A8b	Area 8b
A46D	Area 46, dorsal part
A8aD	Area 8a, dorsal part
A8aV	Area 8a, ventral part
**Ventrolateral prefrontal cortex (vlPFC)**	
A45	Area 45
A47L	Area 47 (12), lateral part
**Orbito frontal cortex (OFC)**	
A11	Area 11
**Medial prefrontal cortex (mPFC)**	
A24d	Area 24d
A32	Area 32
A32V	Area 32, ventral part
**Motor and premotor cortex (MOT)**	
A6M	Area 6, medial (supplementary motor) part
A6Va	Area 6, ventral, part a
A6DC	Area 6, dorsocaudal part
A4ab	Area 4, parts a and b (primary motor, representation of axial and limb musculature)
A4c	Area 4, part c (primary motor, representation of head musculature)
A8C	Area 8, caudal part
A6DR	Area 6, dorsorostral part
**Somatosensory cortex (SS)**	
S2E	Secondary somatosensory area, external part
A1-2	Areas 1 and 2

A3a	Area 3a
A3b	Area 3b (primary somatosensory area)
**Auditory cortex (AU)**	
AuA1	Primary auditory area
AuCPB	Caudal parabelt area
AuRT	Rostrotemporal area
AuCM	Caudomedial area
AuML	Middle lateral area
**Lateral and inferior temporal cortex (LIT)**	
PGa-IPa	Areas PGa and IPa (fundus of superior temporal ventral area)
TPO	Temporo-parieto-occipital association area
TE3	Temporal area TE, part 3
TEO	Temporal area TE, occipital transition part
**Posterior cingulate, medial, and retrosplenial cortex (PCC)**	
A23c	Area 23c
A23b	Area 23b
A23a	Area 23a
**Posterior parietal cortex (PPC)**	
PF	Parietal area PF
PE	Parietal area PE
MIP	Medial intraparietal area
PEC	Parietal area PE, caudal transitional part
AIP	Anterior intraparietal area
PG	Parietal area PG
PFG	Parietal area PFG
PGM	Parietal area PG, medial part
OPt	Occipito-parietal transitional area
LIP	Lateral intraparietal area
**Visual cortex (VC)**	
V1	Primary visual area
V2	Visual area 2
V4	Visual area 4 (ventrolatereral anterior area, VLA)
V4T	Visual area 4, transitional part (middle temporal crescent)

V5	Visual area 5 (middle temporal area, MT)
A19DI	Area 19, dorsointermediate part (DI)
V3A	Visual area 3a (dorsoanterior area, DA)
V6	Visual area 6 (dorsomedial area, DM)
A19M	Area 19, medial part
MST	Medial superior temporal area

Table S2. Details of homologous landmarks between humans and marmosets[32]

#	Homologous area label	Label name in HCP-MMP1 atlases[33]	Label name in Paxinos atlases[7,30]	References
1	V1	V1	V1	Solomon and Rosa[34]
2	V2	V2	V2	Solomon and Rosa[34]
3	4	4	A4ab	Kaas[35]
4	3a	3a	A3a	Kaas[35]
5	3b	3b	A3b	Kaas[35]
6	1	1	A1-2	Kaas[35]
7	8aV	8aV	A8aV	Solomon and Rosa[34]
8	MT	MT	V5	Solomon and Rosa[34]
9	dlPFC	8Ad	A8aD	Liu et al.[1], Ji et al[36]
10	PCC	7m	PGM	Liu et al.[1], Ji et al.[36]
11	PPC	PGi	LIP	Liu et al.[1], Ji et al.[36]

Supplementary References

1. Liu, C. et al. Anatomical and functional investigation of the marmoset default mode network. *Nat. Commun.* **10**, 1975 (2019).
2. Tian, X. et al. An integrated resource for functional and structural connectivity of the marmoset brain. *Nat. Commun.* **13**, 7416 (2022).
3. Cox, R. W. AFNI: software for analysis and visualization of functional magnetic resonance neuroimages. *Comput. biomed. Res.* **29**, 162-173 (1996).
4. Smith, S. M. et al. Advances in functional and structural MR image analysis and implementation as FSL. *NeuroImage* **23**, S208-S219 (2004).
5. Avants, B. B., Tustison, Song, N. et al., Advanced normalization tools (ants), *Insight J* **2,** 1–35 (2009).
6. Marcus, D. S. et al. Informatics and data mining tools and strategies for the human connectome project. *Front. Neuroinformatics* **5**, 4 (2011).
7. Liu, C. et al. Marmoset brain mapping v3: Population multi-modal standard volumetric and surface-based temeplates. *NeuroImage* ***226***, 117620 (2021).
8. Chung, F. Laplacians and the Cheeger inequality for directed graphs. *Ann. Comb.* **9**, 1–19 (2005).
9. Lovász, L. Random walks on graphs: A survey. *Combinatorics*. **2**, 1–46 (1993).
10. Spielman, D. *Spectral graph theory* (CRC Press Boca Raton, Florida, 2012)..
11. Sevi, H., Rilling, G. & Borgnat, P. Harmonic analysis on directed graphs and applications: From Fourier analysis to wavelets. *Appl. Comput. Harmon. Analysis* **62**, 390–440 (2023).
12. Seabrook, E. & Wiskott, L. A tutorial on the spectral theory of Markov chains. *Neural Comput.* **35**, 1713–1796 (2023).
13. Rubinov, M., & Sporns, O. Complex network measures of brain connectivity: uses and interpretations. *NeuroImage* **52**, 1059-1069 (2010).
14. Váša, F., & Mišić, B. Null models in network neuroscience. *Nat. Rev. Neurosci.* **23**, 493-504 (2022).
15. Wagner, H. H. & Dray, S. Generating spatially constrained null models for irregularly spaced data using Moran spectral randomization methods. *Methods Ecol. Evol.* **6**, 1169–1178 (2015).
16. Vos de Wael, R. et al. Brainspace: a toolbox for the analysis of macroscale gradients in neuroimaging and connectomics datasets. *Commun. Biol*. **3**, 103 (2020).
17. Pirondini, E., Vybornova, A., Coscia, M. & Van De Ville, D. A spectral method for generating surrogate graph signals. *IEEE signal process.Lett.* **23**, 1275–1278 (2016)..
18. Huang, W. et al. A graph signal processing perspective on functional brain imaging. *P. IEEE* **106**, 868–885 (2018).
19. Preti, M. G., & Van De Ville, D. Decoupling of brain function from structure reveals regional behavioral specialization in humans. *Nat. Commun.* **10**, 4747 (2019).
20. Margulies, D. S. et al. Situating the default-mode network along a principal gradient of macroscale cortical organization. *Proc. Natl. Acad. Sci. U. S. A.* **113**, 12574–12579 (2016).
21. Yang, S. et al. Cortical patterning of morphometric similarity gradient reveals diverged hierarchical organization in sensory-motor cortices. *Cell Rep.* **36**, 109582 (2021).
22. Meng, Y. et al. Cortical gradient of a human functional similarity network captured by the geometry of cytoarchitectonic organization. *Commun. Biol.* **5**, 1152 (2022).
23. Burt, J. B., Helmer, M., Shinn, M., Anticevic, A. & Murray, J. D. Generative modeling of brain maps with spatial autocorrelation. *NeuroImage* **220**, 117038 (2020).
24. Viladomat, J., Mazumder, R., McInturff, A., McCauley, D. J. & Hastie, T. Assessing the significance of global and local correlations under spatial autocorrelation: a nonparametric approach. *Biometrics* **70**, 409–418 (2014).

25. Gallager, R. G. *Stochastic processes: theory for applications* (Cambridge University Press, 2013).
26. Wielandt, H. Unzerlegbare, nicht negative matrizen. *Math. Zeitschrift* **52**, 642–648 (1950).
27. Hom, R. A. & Johnson, C. R. Matrix analysis. *Camb. Univ. Express* **455** (1985).
28. Patil, K. R., Jung, K. & Eickhoff, S. B. Commentary on pang et al.(2023) Nature. *bioRxiv* Preprint at https://doi.org/10.1101/2023.10.06.561240 (2023).
29. Jones, E. G. Golgi, cajal and the neuron doctrine. *J. Hist. Neurosci.* **8**, 170–178 (1999).
30. Paxinos, W. C. P. M. R. M. T. H., G. *The Marmoset Brain in Stereotaxic Coordinates* (New York:Academic Press, 2012)..
31. Majka, P. et al. Open access resource for cellular-resolution analyses of corticocortical connectivity in the marmoset monkey. *Nat. commun.* **11**, 1133 (2020).
32. Ngo, G. N., Hori, Y., Everling, S. & Menon, R. S. Joint-embeddings reveal functional differences in default-mode network architecture between marmosets and humans. *NeuroImage* **272**, 120035 (2023)..
33. Glasser, M. F. et al. A multi-modal parcellation of human cerebral cortex. *Nature* **536**, 171–178 (2016).
34. Solomon, S. G., & Rosa, M. G. A simpler primate brain: the visual system of the marmoset monkey. *Front. Neural Circuits.* **8**, 96 (2014).
35. Kaas, J. H. Evolution of somatosensory and motor cortex in primates. *Anat. Rec.* **281**, 1148-1156 (2004).
36. Ji, J. L. et al. Mapping the human brain's cortical-subcortical functional network organization. *NeuroImage* **185**, 35-57 (2019).
