# Supplementary material for: Decomposing cortical activity through neuronal tracing connectome-eigenmodes in marmosets
Source: Nat Commun. 2024 Mar 13;15:2289. doi: 10.1038/s41467-024-46651-8 (PMC10937940; doi:10.1038/s41467-024-46651-8)
Supplement: Supplementary file 3 — Reporting Summary [file 41467_2024_46651_MOESM3_ESM.pdf]

nature portfolio

Corresponding author(s): Huafu CHEN; Wei LIAO

Last updated by author(s): Mar 1, 2024

Reporting Summary

Nature Portfolio wishes to improve the reproducibility of the work that we publish. This form provides structure for consistency and transparency in reporting. For further information on Nature Portfolio policies, see our Editorial Policies and the Editorial Policy Checklist.

Statistics

For all statistical analyses, confirm that the following items are present in the figure legend, table legend, main text, or Methods section.

n/a	Confirmed	
[ ]	[x]	The exact sample size (n) for each experimental group/condition, given as a discrete number and unit of measurement
[ ]	[x]	A statement on whether measurements were taken from distinct samples or whether the same sample was measured repeatedly
[ ]	[x]	The statistical test(s) used AND whether they are one- or two-sided<br>*Only common tests should be described solely by name; describe more complex techniques in the Methods section.*
[ ]	[x]	A description of all covariates tested
[ ]	[x]	A description of any assumptions or corrections, such as tests of normality and adjustment for multiple comparisons
[ ]	[x]	A full description of the statistical parameters including central tendency (e.g. means) or other basic estimates (e.g. regression coefficient) AND variation (e.g. standard deviation) or associated estimates of uncertainty (e.g. confidence intervals)
[ ]	[x]	For null hypothesis testing, the test statistic (e.g. F, t, r) with confidence intervals, effect sizes, degrees of freedom and P value noted<br>*Give P values as exact values whenever suitable.*
[x]	[ ]	For Bayesian analysis, information on the choice of priors and Markov chain Monte Carlo settings
[x]	[ ]	For hierarchical and complex designs, identification of the appropriate level for tests and full reporting of outcomes
[ ]	[x]	Estimates of effect sizes (e.g. Cohen's d, Pearson's r), indicating how they were calculated

Our web collection on statistics for biologists contains articles on many of the points above.

Software and code

Policy information about availability of computer code

Data collection	All MRI marmoset data were collected using Bruker's ParaVision V.6.0.1.
Data analysis	The neuroimaging preprocessing software is freely available (AFNI v18.0.11, https://afni.nimh.nih.gov/, FSL v5.1, https://fsl.fmrib.ox.ac.uk/fsl/fslwiki, ANTs v2.1, https://picsl.upenn.edu/software/ants/, Connectome Workbench v1.5.0, https://www.humanconnectome.org/software/connectome-workbench, and MRtrix3 v3, https://www.mrtrix.org/).<br>The code of resting state fMRI data processing is openly available at https://marmosetbrainmapping.org/v4_readme.html.<br>The code used to conduct the main results in this study is available at https://github.com/weiliao81/Marmoset_CFD and on Zenodo (https://doi.org/10.5281/ zenodo.10728317). The code for spatial autocorrelation-preserving surrogate brain maps can be implemented through the brainSMASH toolbox (https://github.com/murraylab/brainsmash). The code for gradient analysis and the Moran spectral randomization can be performed via BrainSpace (http://github.com/MICA-MNI/BrainSpace). The brain surfaces were visualized using Connectome Workbench (v1.5.0, https://www.humanconnectome.org/software/ connectome-workbench) and Python script (https://github.com/netneurolab/marmoset_connectome).

For manuscripts utilizing custom algorithms or software that are central to the research but not yet described in published literature, software must be made available to editors and reviewers. We strongly encourage code deposition in a community repository (e.g. GitHub). See the Nature Portfolio guidelines for submitting code & software for further information.

Data

Policy information about availability of data

All manuscripts must include a data availability statement. This statement should provide the following information, where applicable:

- Accession codes, unique identifiers, or web links for publicly available datasets
- A description of any restrictions on data availability
- For clinical datasets or third party data, please ensure that the statement adheres to our policy

The retrograde neuroanatomical tract-tracing data is publicly available from the Marmoset Brain Architecture Project (http://marmoset.braincircuits.org/). The NIH and ION awake marmosets MRI datasets are available from the Marmoset Brain Mapping (https://marmosetbrainmapping.org/data).
The HCP dataset is publicly available at https://db.humanconnectome.org/.
The Paxinos marmoset parcellation is publicly available from the MBMv3 resource (https://marmosetbrainmapping.org/v3.html).
The HCP-MMP1.0 human cortical atlas61 is publicly available at https://balsa.wustl.edu/study/show/RVVG.
The myelin content, allometric scale, and cortical gene expression in human is available through neuromaps (https://github.com/netneurolab/neuromaps). Source data are provided with this paper.

Research involving human participants, their data, or biological material

Policy information about studies with human participants or human data. See also policy information about sex, gender (identity/presentation), and sexual orientation and race, ethnicity and racism.

Reporting on sex and gender	For the human participants, 54 of 100 reported their sex as female and 46 as male. No additional information on gender was available.
Reporting on race, ethnicity, or other socially relevant groupings	This information was not available. See https://db.humanconnectome.org/app/template/SubjectDashboard.vm?project=HCP_1200&subjectGroupName=100%20Unrelated%20Subjects
Population characteristics	A sample of 100 unrelated healthy subjects (54% female; mean age = 29.1±3.7 years; age range = 22-36 years) was drawn from the HCP dataset, as publicly provided by the HCP1200 subjects data release.
Recruitment	A sample of 100 unrelated healthy subjects was drawn from the HCP dataset, as publicly provided by the HCP1200 subjects data release.
Ethics oversight	Informed consent was obtained from all Human Connectome Project subjects, and the procedures were approved by the Washington University Institutional Review Board. Informed consent was obtained from all subjects scanned at the University of Pennsylvania, and the procedures were approved by the University of Pennsylvania Institutional Review Board. All participants were volunteers and provided informed consent.

Note that full information on the approval of the study protocol must also be provided in the manuscript.

Field-specific reporting

Please select the one below that is the best fit for your research. If you are not sure, read the appropriate sections before making your selection.

☒ Life sciences ☐ Behavioural & social sciences ☐ Ecological, evolutionary & environmental sciences

For a reference copy of the document with all sections, see nature.com/documents/nr-reporting-summary-flat.pdf

Life sciences study design

All studies must disclose on these points even when the disclosure is negative.

Sample size	The current study utilized the awake marmoset resting-state fMRI data from two independents institutes (total n = 19). No statistical methods were used to pre-determine sample size.The sample size is adequate to address our scientific questions, because the population-level cellular-functional relationship produced highly similar results between ION and NIH datasets. The aim is to report consistent results across subjects. No subject-specific features or traits are used.
Data exclusions	For each fMRI run, any time points and the previous time points were censored if the detection motion was >0.2 mm. The motion censor iscommonly used in fMRI preprocess to reduce the influence of head motions. No data were excluded from the analysis.
Replication	We include sensitivity and reproducibility assessments in the manuscript. We then conducted a spatial correlation of the CFD pattern between the NIH and ION site datasets. The results showed a statistically significant positive correlation (ρ = 0.83, pSMASH = 0.0001). Furthermore, the Dice coefficient was 0.73 and 0.70 for significantly decoupled and coupled areas, respectively. As a result, these findings confirmed the reproducibility of the CFD patterns.

Randomization	Randomization was not relevant to our study. There are no experimental and control groups in our study. All marmoset and human resting state fMRI data were single groups.
Blinding	There are no experimental and control groups in our study. All marmoset and human resting state fMRI data were normal subjects and belonged to one health group.

Behavioural & social sciences study design

All studies must disclose on these points even when the disclosure is negative.

Study description	*Briefly describe the study type including whether data are quantitative, qualitative, or mixed-methods (e.g. qualitative cross-sectional, quantitative experimental, mixed-methods case study).*
Research sample	*State the research sample (e.g. Harvard university undergraduates, villagers in rural India) and provide relevant demographic information (e.g. age, sex) and indicate whether the sample is representative. Provide a rationale for the study sample chosen. For studies involving existing datasets, please describe the dataset and source.*
Sampling strategy	*Describe the sampling procedure (e.g. random, snowball, stratified, convenience). Describe the statistical methods that were used to predetermine sample size OR if no sample-size calculation was performed, describe how sample sizes were chosen and provide a rationale for why these sample sizes are sufficient. For qualitative data, please indicate whether data saturation was considered, and what criteria were used to decide that no further sampling was needed.*
Data collection	*Provide details about the data collection procedure, including the instruments or devices used to record the data (e.g. pen and paper, computer, eye tracker, video or audio equipment) whether anyone was present besides the participant(s) and the researcher, and whether the researcher was blind to experimental condition and/or the study hypothesis during data collection.*
Timing	*Indicate the start and stop dates of data collection. If there is a gap between collection periods, state the dates for each sample cohort.*
Data exclusions	*If no data were excluded from the analyses, state so OR if data were excluded, provide the exact number of exclusions and the rationale behind them, indicating whether exclusion criteria were pre-established.*
Non-participation	*State how many participants dropped out/declined participation and the reason(s) given OR provide response rate OR state that no participants dropped out/declined participation.*
Randomization	*If participants were not allocated into experimental groups, state so OR describe how participants were allocated to groups, and if allocation was not random, describe how covariates were controlled.*

Ecological, evolutionary & environmental sciences study design

All studies must disclose on these points even when the disclosure is negative.

Study description	*Briefly describe the study. For quantitative data include treatment factors and interactions, design structure (e.g. factorial, nested, hierarchical), nature and number of experimental units and replicates.*
Research sample	*Describe the research sample (e.g. a group of tagged Passer domesticus, all Stenocereus thurberi within Organ Pipe Cactus National Monument), and provide a rationale for the sample choice. When relevant, describe the organism taxa, source, sex, age range and any manipulations. State what population the sample is meant to represent when applicable. For studies involving existing datasets, describe the data and its source.*
Sampling strategy	*Note the sampling procedure. Describe the statistical methods that were used to predetermine sample size OR if no sample-size calculation was performed, describe how sample sizes were chosen and provide a rationale for why these sample sizes are sufficient.*
Data collection	*Describe the data collection procedure, including who recorded the data and how.*
Timing and spatial scale	*Indicate the start and stop dates of data collection, noting the frequency and periodicity of sampling and providing a rationale for these choices. If there is a gap between collection periods, state the dates for each sample cohort. Specify the spatial scale from which the data are taken*
Data exclusions	*If no data were excluded from the analyses, state so OR if data were excluded, describe the exclusions and the rationale behind them, indicating whether exclusion criteria were pre-established.*
Reproducibility	*Describe the measures taken to verify the reproducibility of experimental findings. For each experiment, note whether any attempts to repeat the experiment failed OR state that all attempts to repeat the experiment were successful.*
Randomization	*Describe how samples/organisms/participants were allocated into groups. If allocation was not random, describe how covariates were controlled. If this is not relevant to your study, explain why.*

Blinding | *Describe the extent of blinding used during data acquisition and analysis. If blinding was not possible, describe why OR explain why blinding was not relevant to your study.*

Did the study involve field work? ☐ Yes ☒ No

Reporting for specific materials, systems and methods

We require information from authors about some types of materials, experimental systems and methods used in many studies. Here, indicate whether each material, system or method listed is relevant to your study. If you are not sure if a list item applies to your research, read the appropriate section before selecting a response.

Materials & experimental systems

n/a	Involved in the study
☒	☐ Antibodies
☒	☐ Eukaryotic cell lines
☒	☐ Palaeontology and archaeology
☐	☒ Animals and other organisms
☒	☐ Clinical data
☒	☐ Dual use research of concern
☒	☐ Plants

Methods

n/a	Involved in the study
☒	☐ ChIP-seq
☒	☐ Flow cytometry
☐	☒ MRI-based neuroimaging

Antibodies

Antibodies used | *Describe all antibodies used in the study; as applicable, provide supplier name, catalog number, clone name, and lot number.*

Validation | *Describe the validation of each primary antibody for the species and application, noting any validation statements on the manufacturer's website, relevant citations, antibody profiles in online databases, or data provided in the manuscript.*

Eukaryotic cell lines

Policy information about cell lines and Sex and Gender in Research

Cell line source(s) | *State the source of each cell line used and the sex of all primary cell lines and cells derived from human participants or vertebrate models.*

Authentication | *Describe the authentication procedures for each cell line used OR declare that none of the cell lines used were authenticated.*

Mycoplasma contamination | *Confirm that all cell lines tested negative for mycoplasma contamination OR describe the results of the testing for mycoplasma contamination OR declare that the cell lines were not tested for mycoplasma contamination.*

Commonly misidentified lines (See ICLAC register) | *Name any commonly misidentified cell lines used in the study and provide a rationale for their use.*

Palaeontology and Archaeology

Specimen provenance | *Provide provenance information for specimens and describe permits that were obtained for the work (including the name of the issuing authority, the date of issue, and any identifying information). Permits should encompass collection and, where applicable, export.*

Specimen deposition | *Indicate where the specimens have been deposited to permit free access by other researchers.*

Dating methods | *If new dates are provided, describe how they were obtained (e.g. collection, storage, sample pretreatment and measurement), where they were obtained (i.e. lab name), the calibration program and the protocol for quality assurance OR state that no new dates are provided.*

☐ Tick this box to confirm that the raw and calibrated dates are available in the paper or in Supplementary Information.

Ethics oversight | *Identify the organization(s) that approved or provided guidance on the study protocol, OR state that no ethical approval or guidance was required and explain why not.*

Note that full information on the approval of the study protocol must also be provided in the manuscript.

Animals and other research organisms

Policy information about studies involving animals; ARRIVE guidelines recommended for reporting animal research, and Sex and Gender in Research

Laboratory animals	We recruited 21 common marmosets (Callithrix jacchus, in age ranges of 2 to 9 year old). Thirteen marmosets (12 males and 1 female) were recruited from the ION cohort and seven marmosets (7 males) were recruited from the NIH cohort.
Wild animals	This study did not involve wild animals.
Reporting on sex	NIH cohort: 7 males; ION cohort: 12 males and 1 female.
Field-collected samples	The study did not involve samples collected from the field.
Ethics oversight	The experimental procedures were approved by the National Institute of Neurological Disorders and Stroke at the National Institutes ofHealth (NIH) and Laboratory Animal Care and Use Committees from the Institute of Neuroscience (ION) at the Chinese Academy of Sciences.

Note that full information on the approval of the study protocol must also be provided in the manuscript.

Clinical data

Policy information about clinical studies

All manuscripts should comply with the ICMJEguidelines for publication of clinical research and a completedCONSORT checklist must be included with all submissions.

Clinical trial registration	*Provide the trial registration number from ClinicalTrials.gov or an equivalent agency.*
Study protocol	*Note where the full trial protocol can be accessed OR if not available, explain why.*
Data collection	*Describe the settings and locales of data collection, noting the time periods of recruitment and data collection.*
Outcomes	*Describe how you pre-defined primary and secondary outcome measures and how you assessed these measures.*

Dual use research of concern

Policy information about dual use research of concern

Hazards

Could the accidental, deliberate or reckless misuse of agents or technologies generated in the work, or the application of information presented in the manuscript, pose a threat to:

No	Yes	
☒	☐	Public health
☒	☐	National security
☒	☐	Crops and/or livestock
☒	☐	Ecosystems
☒	☐	Any other significant area

Experiments of concern

Does the work involve any of these experiments of concern:

No	Yes	
☒	☐	Demonstrate how to render a vaccine ineffective
☒	☐	Confer resistance to therapeutically useful antibiotics or antiviral agents
☒	☐	Enhance the virulence of a pathogen or render a nonpathogen virulent
☒	☐	Increase transmissibility of a pathogen
☒	☐	Alter the host range of a pathogen
☒	☐	Enable evasion of diagnostic/detection modalities
☒	☐	Enable the weaponization of a biological agent or toxin
☒	☐	Any other potentially harmful combination of experiments and agents

Plants

Seed stocks	*Report on the source of all seed stocks or other plant material used. If applicable, state the seed stock centre and catalogue number. If plant specimens were collected from the field, describe the collection location, date and sampling procedures.*
Novel plant genotypes	*Describe the methods by which all novel plant genotypes were produced. This includes those generated by transgenic approaches, gene editing, chemical/radiation-based mutagenesis and hybridization. For transgenic lines, describe the transformation method, the number of independent lines analyzed and the generation upon which experiments were performed. For gene-edited lines, describe the editor used, the endogenous sequence targeted for editing, the targeting guide RNA sequence (if applicable) and how the editor was applied.*
Authentication	*Describe any authentication procedures for each seed stock used or novel genotype generated. Describe any experiments used to assess the effect of a mutation and, where applicable, how potential secondary effects (e.g. second site T-DNA insertions, mosiacism, off-target gene editing) were examined.*

ChIP-seq

Data deposition

☐ Confirm that both raw and final processed data have been deposited in a public database such as GEO.

☐ Confirm that you have deposited or provided access to graph files (e.g. BED files) for the called peaks.

Data access links<br>*May remain private before publication.*	*For "Initial submission" or "Revised version" documents, provide reviewer access links. For your "Final submission" document, provide a link to the deposited data.*
Files in database submission	*Provide a list of all files available in the database submission.*
Genome browser session<br>(e.g. UCSC)	*Provide a link to an anonymized genome browser session for "Initial submission" and "Revised version" documents only, to enable peer review. Write "no longer applicable" for "Final submission" documents.*

Methodology

Replicates	*Describe the experimental replicates, specifying number, type and replicate agreement.*
Sequencing depth	*Describe the sequencing depth for each experiment, providing the total number of reads, uniquely mapped reads, length of reads and whether they were paired- or single-end.*
Antibodies	*Describe the antibodies used for the ChIP-seq experiments; as applicable, provide supplier name, catalog number, clone name, and lot number.*
Peak calling parameters	*Specify the command line program and parameters used for read mapping and peak calling, including the ChIP, control and index files used.*
Data quality	*Describe the methods used to ensure data quality in full detail, including how many peaks are at FDR 5% and above 5-fold enrichment.*
Software	*Describe the software used to collect and analyze the ChIP-seq data. For custom code that has been deposited into a community repository, provide accession details.*

Flow Cytometry

Plots

Confirm that:

☐ The axis labels state the marker and fluorochrome used (e.g. CD4-FITC).

☐ The axis scales are clearly visible. Include numbers along axes only for bottom left plot of group (a 'group' is an analysis of identical markers).

☐ All plots are contour plots with outliers or pseudocolor plots.

☐ A numerical value for number of cells or percentage (with statistics) is provided.

Methodology

Sample preparation	*Describe the sample preparation, detailing the biological source of the cells and any tissue processing steps used.*
Instrument	*Identify the instrument used for data collection, specifying make and model number.*
Software	*Describe the software used to collect and analyze the flow cytometry data. For custom code that has been deposited into a community repository, provide accession details.*

Cell population abundance	*Describe the abundance of the relevant cell populations within post-sort fractions, providing details on the purity of the samples and how it was determined.*
Gating strategy	*Describe the gating strategy used for all relevant experiments, specifying the preliminary FSC/SSC gates of the starting cell population, indicating where boundaries between "positive" and "negative" staining cell populations are defined.*

☐ Tick this box to confirm that a figure exemplifying the gating strategy is provided in the Supplementary Information.

Magnetic resonance imaging

Experimental design

Design type	Resting state fMRI (marmoset and human) and diffusion MRI (marmoset).
Design specifications	Marmoset Brain Architecture Project dataset:<br>All rs-fMRI data were scanned using a 2D gradient echo planar imaging (EPI) sequence (repetition time (TR) = 2s, 512 volumes (17 min) per run). After each rs-fMRI session, a T2-weighted image was scanned for spatial registration. Furthermore, in vivo diffusion MRI (dMRI) data were acquired using a 2D diffusion-weighted spin-echo EPI sequence.<br>Human Connectome Project dataset:<br>Resting-state fMRI (gradient-echo echo-planar imaging [EPI] sequence; four runs; 1200 volumes/run, 14:33 min:sec each; voxel size: 2 mm isotropic; repetition time [TR]: 720 ms; echo time [TE]: 33.1 ms).
Behavioral performance measures	n/a (resting-state fMRI did not involve behavioral performance measures).

Acquisition

Imaging type(s)	Marmoset: Functional (resting state fMRI); Structural (T2-weighed anatomical image and diffusion MRI).<br>Human: Functional (resting state fMRI); Structural (T1-weighed anatomical image).
Field strength	Marmoset: (NIH) 7T, Bruker; (ION) 9.4T, Bruker<br>Human: 3T, Siemens
Sequence & imaging parameters	The acquisition parameters of awake marmoset rs-fMRI were described in details by the Marmoset Brain Mapping Project.<br>The NIH marmosets were scanned in a 7T/30cm horizontal MRI (Bruker, Billerica, USA) equipped with a 15 cm customized gradient set capable of 450 mT/m gradient strength (Resonance Research Inc.,Billerica, USA). Resting-state fMRI (rs-fMRI) data were acquired with a 2D gradient echo planar imaging (EPI) sequence (TR = 2000 ms, TE = 22.2 ms, flip angle = 70.4°, FOV = 28 × 36 mm2, matrix size = 56 × 72, 38 axial slices, resolution = 0.5 mm isotropic, 512 time points, each run is 17 min long). Two sets of spin-echo EPI with opposite phase-encoding directions (LR and RL) were also collected for EPI-distortion correction (TR = 3000 ms, TE = 36 ms, flip angle = 90°, FOV = 28 × 36 mm°, matrix size = 56 × 72, 38 axial slices, slice thickness = 0.5 mm, 8 volumes for each set). For each session, a T2-weighted structural image was scanned for spatial registration (TR = 6000 ms, TE = 9 ms, flip angle = 90°, FOV = 28 × 36 mm2, matrix size = 112 × 144, 38 axis slices, slice thickness=0.5 mm). Furthermore, multishell diffusion-weighted imaging (DWI) were collected using a 2D spin-echo EPI sequence as follows: TR = 5100 ms, TE = 38 ms, a number of segments = 88, FOV = 36 × 28 mm2, matrix size = 72 × 56, slice thickness = 0.5 mm, a total of 400 DWI images for two-phase encodings (blip-up and blip-down) and each has 3 b values (8 b= 0, 64 b = 2400, and 128b= 4800), and the scanning duration was ~34 min.<br>The ION marmosets were scanned in a 9.4T/30cm horizontal MRI scanner (Bruker,Billerica,USA) equipped with a 20 cm gradient set capable of 300 mT/m gradient strength.The scanner was fitted with a 154 mm ID quadrature RF coil used for signal excitation and an 8-channel phased-array RF coil custom-built for marmosets (Fine Instrument Technology,Brazil). Multiple runs of rs-fMRIl data were collected in ParaVision 6.0.1 software using a 2D gradient-echo (GE) EPI sequence with the following parameters: TR=2S, TE=18 ms, flip angle=70.4°, FOV=28×36mm, matrix size=56×72,38axial slices, slice thickness=0.5 mm, 512 volumes (17min) per run. The GE-EPI fMRI data were collected using two opposite phase-encoding directions(LR and RL) to compensate for EPI distortions and signal dropouts. Two sets of spin-echo EPI with opposite phase-encoding directions (LR and RL) were also collected for EPI-distortion correction (TR=3000ms,TE=37.69 ms, flip angle=90°,FOV=28x36 mm, matrix size=56×72,38 axial slices, slice thickness=0.5 mm, 8volumes for each set). After each rs-fMRI session, a T2-weighted structural image (TR=8000 ms,TE=10 ms, flip angle=90°,FOV=28×36mm, matrix size=112×144,38 axial slices, slice thickness=0.5mm) was scanned for co-registration purposes.<br>Human Connectome Project dataset:<br>Subjects within this sample were scanned on a customized Siemens "Connectome" Skyra 3T scanner (32-channel Siemens head coil) and underwent high-resolution 3T MRI, including T1-weighted (3D Multi-echo Magnetization–Prepared Rapid Gradient Echo [MEMPRAGE] sequence; voxel size: 0.7 mm isotropic; TR: 2400 ms; TE: 2.14 ms), T2-weighted (3D sampling perfection with application-optimized contrasts by using flip angle evolution [SPACE] sequence; voxel size: 0.7 mm isotropic; TR: 3200 ms; TE: 565 ms), resting-state fMRI (gradient-echo echo-planar imaging [EPI] sequence; four runs; 1200 volumes/run, 14:33 min:sec each; voxel size: 2 mm isotropic; TR: 720 ms; TE: 33.1 ms), and high angular resolution diffusion imaging (spin-echo planar imaging sequence; voxel size: 1.25 mm isotropic; TR: 5520 ms; TE: 89.5 ms; max b-value: 3000 s/mm2; 270 non-colinear directions; 18 b0 acquisitions) sequences.
Area of acquisition	Whole brain

Diffusion MRI ☒ Used ☐ Not used

Preprocessing

Preprocessing software	Preprocessing involves the following software:<br>AFNI v18.0.11, https://afni.nimh.nih.gov/,<br>FSL v5.1, https://fsl.fmrib.ox.ac.uk/fsl/fslwiki,<br>ANTs v2.1, https://picsl.upenn.edu/software/ants/,<br>Connectome Workbench 1.5.0, https://www.humanconnectome.org/software/connectome-workbench,<br>and MRtrix3 v3, https://www.mrtrix.org/
Normalization	The preprocessed data were spatially normalized to the template space of Marmoset Brain Atlas Version-3 (MBM3) by the "antsRegistration" routine of ANTs. The spatial normalization concatenated multiple transformations, including 1) rigid-body transformation of each fMRI run to the T2-weighted image acquired at the end of each session, 2) rigid-body transformation of T2-weighted images from each session to a cross-session averaged T2-weighted image from each animal,3) affine and nonlinear transformation of the averaged T2-weighted image from each animal to the T2w template of MBMv3 space.
Normalization template	We normalized the fMRI data to the MBMv3 template space.
Noise and artifact removal	The rs-fMRI datasets were further preprocessed by regressing linear and quadratic trends, demeaning, and censoring for motion using derivatives of motion parameters and motion-sensor regressors (any TRs and the previous TRs were censored ifthe detection motion was > 0.2mm).white matter and cerebrospinal fluid signal were removed, and the rs-fMRI datasets were band-pass filtered (0.01-0.1Hz).The above nuisance signal regression and band-passing fltering were carried out bythe "3dDeconvolve" and "3dTproject" commands in AFNI.
Volume censoring	No fMRI volume was censored during fMRI preprocessing.

Statistical modeling & inference

Model type and settings	General linear model performed only for nuisance regression.
Effect(s) tested	Not relevant to this study.

Specify type of analysis: ☐ Whole brain ☐ ROI-based ☒ Both

Statistic type for inference<br>(See Eklund et al. 2016)	We performed a nonparametric permutation test to examine the spatial significance of the cortical classes' level energy concentrations (see Statistical analysis).
Correction	See Methods and above for descriptions on the multiple permutation strategies employed to test robustness of empirical effects.

Models & analysis

n/a	Involved in the study
☐	☒ Functional and/or effective connectivity
☐	☒ Graph analysis
☒	☐ Multivariate modeling or predictive analysis

Functional and/or effective connectivity	Functional connectivity was measured as inter-regional correlations (Pearson's correlation).
Graph analysis	The weighted directed graph was build using the retrograde neuroanatomical tract-tracing data in marmosets. The strength of the tract-tracing cellular connectome was quantified from the fraction of extrinsic labeled neurons.
Multivariate modeling and predictive analysis	*Specify independent variables, features extraction and dimension reduction, model, training and evaluation metrics.*
